# No association between *ACTN3* R577X and *ACE* I/D polymorphisms and endurance running times in 698 Caucasian athletes

**DOI:** 10.1186/s12864-017-4412-0

**Published:** 2018-01-03

**Authors:** Ioannis D. Papadimitriou, Sarah J. Lockey, Sarah Voisin, Adam J. Herbert, Fleur Garton, Peter J. Houweling, Pawel Cieszczyk, Agnieszka Maciejewska-Skrendo, Marek Sawczuk, Myosotis Massidda, Carla Maria Calò, Irina V. Astratenkova, Anastasia Kouvatsi, Anastasiya M. Druzhevskaya, Macsue Jacques, Ildus I. Ahmetov, Georgina K. Stebbings, Shane Heffernan, Stephen H. Day, Robert Erskine, Charles Pedlar, Courtney Kipps, Kathryn N. North, Alun G. Williams, Nir Eynon

**Affiliations:** 1 Institute of Sport, Exercise and Active Living (ISEAL), Victoria University, Victoria, Australia; 2 Sports Genomics Laboratory, Manchester Metropolitan University, Crewe, UK; 3 Institute for Molecular Bioscience, University of Queensland, Queensland, Australia; 4 Murdoch Children’s Research Institute, Melbourne, Australia; 5 Faculty of Physical Education, Gdansk University of Physical Education and Sport, Gdansk, Poland; 6 Faculty of Tourism and Recreation, Gdansk University of Physical Education and Sport, Gdańsk, Poland; 7 Department of Life and Environmental Sciences, University of Cagliari, Cagliari, Italy; 8 Sports Genetics Laboratory, St Petersburg Research Institute of Physical Culture, St Petersburg, Russia; 9 Department of Genetics Development and Molecular Biology, Aristotle University of Thessaloniki, Thessaloniki, Greece; 10 Laboratory of Molecular Genetics, Kazan State Medical University, Kazan, Russia; 11 Research Institute for Sport and Exercise Sciences, Liverpool John Moores University, Liverpool, UK; 12 School of Sport, Health and Applied Science, St Mary’s University College, Twickenham, UK; 13 Department of Paediatrics, University of Melbourne, Victoria, Australia; 14 Institute of Sport, Exercise and Health, University College London, London, UK

**Keywords:** ACTN3, ACE, Genomics, Athletic performance, Endurance, Champions

## Abstract

**Background:**

Studies investigating associations between *ACTN3* R577X and *ACE* I/D genotypes and endurance athletic status have been limited by small sample sizes from mixed sport disciplines and lack quantitative measures of performance. Aim: To examine the association between *ACTN3* R577X and *ACE* I/D genotypes and best personal running times in a large homogeneous cohort of endurance runners.

**Methods:**

We collected a total of 1064 personal best 1500, 3000, 5000 m and marathon running times of 698 male and female Caucasian endurance athletes from six countries (Australia, Greece, Italy, Poland, Russia and UK). Athletes were genotyped for *ACTN3* R577X and *ACE* ID variants.

**Results:**

There was no association between *ACTN3* R577X or *ACE* I/D genotype and running performance at any distance in men or women. Mean (SD) marathon times (in s) were for men: *ACTN3* RR 9149 (593), RX 9221 (582), XX 9129 (582) *p* = 0.94; *ACE* DD 9182 (665), ID 9214 (549), II 9155 (492) *p* = 0.85; for women: *ACTN3* RR 10796 (818), RX 10667 (695), XX 10675 (553) *p* = 0.36; *ACE* DD 10604 (561), ID 10766 (740), II 10771 (708) *p* = 0.21. Furthermore, there were no associations between these variants and running time for any distance in a sub-analysis of athletes with personal records within 20% of world records.

**Conclusions:**

Thus, consistent with most case-control studies, this multi-cohort quantitative analysis demonstrates it is unlikely that *ACTN3* XX genotype provides an advantage in competitive endurance running performance. For *ACE* II genotype, some prior studies show an association but others do not. Our data indicate it is also unlikely that *ACE* II genotype provides an advantage in endurance running.

## Background

Although the likelihood of becoming an elite athlete is probably influenced by genetic variations across the human genome [1, 2], there is currently no evidence for a common genetic profile specific to elite endurance athletes, even when utilising a Genome-Wide Association Study (GWAS) approach [3]. However, there is considerable evidence suggesting that *ACTN3* R577X and *ACE* I/D gene variants do influence muscle performance and metabolism in humans [4].

A common null polymorphism (rs1815739) was identified in the *ACTN3* gene, which results in the replacement of an arginine (R) residue with a premature stop codon (X) at amino acid 577. Approximately 18% of the world population (~1.5 billion individuals) harbour the *ACTN3* 577XX genotype and consequently are completely deficient in α-actinin-3 protein. Importantly, α-actinin-3 deficiency does not cause any obvious muscle disease [5].

An association between the *ACTN3* R577X genotype and athletic performance was initially found in a cohort of elite Australian athletes [6], with a very low proportion of elite sprint/power athletes harbouring the 577XX genotype. This genotype distribution pattern was quite consistent in other independent cohorts of elite athletes and has since been replicated in Finnish [7], Greek [8], Russian [9], Israeli [10], Polish [11] and Japanese [12] athletes.

A tendency for a higher proportion of elite athletes carrying the 577XX genotype was also found in Australian athletes excelling in aerobic activities [6], showing some evidence for association of this genotype with endurance performance. While this association was replicated in some cohorts of athletes [10, 13] other studies have shown no association between the *ACTN3* R577X genotypes and endurance athletic status [7, 8]. Furthermore, a large study with Russian endurance athletes found that the frequency of the XX genotype was lower in endurance athletes than in controls [14], demonstrating the conflicting results between the association of this gene variant and endurance athletic performance. In line with this finding, an analysis comparing 50 elite male endurance cyclists and 52 Olympic-level endurance runners with 123 sedentary male controls [15] found no difference in genotype frequencies between controls and either of the two athlete groups. There was also no association between R577X genotypes and a common measure of endurance performance - maximal oxygen uptake (VO_2max_) - in either of the athlete groups. Cross-sectional studies showed no association of *ACTN3 XX* genotype with endurance performance [15, 16] as well, and debate is ongoing on whether the *ACTN3* gene influences endurance performance. In a different human sporting context, namely the team sport of rugby union, the R allele has recently been associated with success in playing positions reliant on sprinting speed, while the X allele was associated with playing demands allowing relatively short recovery times [17].

Another candidate gene associated with elite performance is the *ACE* I/D polymorphism. The absence (deletion allele, D) rather than the presence (insertion allele, I) of a 287 base pair fragment is associated with higher tissue [18] and serum [19] ACE activity. While not directly functional [20], the *ACE* I/D polymorphism is related to ACE activity and accounts for up to 40% of the variation in circulating ACE activity in Caucasians [19]. An association between the *ACE* I/D polymorphism and athletic performance was initially found in a cohort of British mountaineers [21], with a very a low proportion of elite mountaineers harbouring the *ACE* DD genotype. This genotype distribution pattern was replicated in cohorts of elite endurance athletes [22, 23]. However, conflicting data also exist with *ACE* I/D genotype and endurance performance [24, 25] also found no association between the *ACE* I/D genotype with VO_2max_ or its response to a 20-week endurance training programme in the HERITAGE Family study. Nevertheless, a more recent meta-analysis concluded that *ACE* II genotype was associated with superior endurance performance, with an odds ratio of 1.35 [4], however this was not replicated in the GAMES GWAS cohort analysis [3].

One of the limitations of most of the abovementioned studies investigating the association between the *ACTN3* R577X and the *ACE* I/D genotypes and athletic status is the grouping of endurance athletes from mixed sport disciplines and events (e.g. middle distance runners, long distance runners, cyclists, swimmers), or analysing team sport athletes from a single sport yet with some variations in physiological demand according to playing position [17]. These approaches, while understandable given the very low number of World-class competitors in a single sport or event, reduce the consistency of the phenotype. Furthermore, those studies only used a simple case-control design based on athletic status without looking at measurable (quantitative) traits within the compared groups [26] and no studies have quantitatively linked those genotypes with endurance performance (e.g. running times) in elite athletes.

We sought to address these limitations by providing deeper insight into the possible association between the *ACTN3* R577X and the *ACE* I/D variants and endurance performance. In the present study, we used the same quantitative approach previously introduced in elite sprinters [27] that showed both *ACTN3* R577X and *ACE I/D* genotypes have a substantial association with sprint performance (100-400 m run) at the elite level. The aim of this study was to examine the association between the *ACTN3* R577X and *ACE I/D* variants and personal best running times in 1500 m, 3000 m, 5000 m, 10,000 m and marathon in a large cohort of male and female Caucasian endurance runners.

## Methods

The methodology been used in genotyping, data collection and statistical analysis has been previously described [17, 27].

### Participants

A total of 1064 personal best 1500, 3000, 5000, 10,000 m and marathon running times of 698 Caucasian endurance athletes (441 males and 257 females) from Australia (*n* = 14), Greece (*n* = 16), Italy (*n* = 9), Poland (*n* = 60), Russia (*n* = 17) and the UK (*n* = 582), were analysed (Table 1). The endurance runners’ personal best times in official competitions were found online (www.iaaf.org and www.thepowerof10.info) or provided by coaches or the athletes themselves and independently corroborated.

**Table 1:** Mean (SD) 1500 m, 3000 m, 5000 m, 10,000 m and marathon best running times in (a) males and (b) females in the three *ACTN3* R577X genotypes

(a)					
*ACTN3*R577X males	RR *N* = 380 34.7%	RX *N* = 492 44.9%	XX *N* = 224 20.4%	Additive (RR = 0, RX = 1, XX = 2)	Recessive (RR = RX = 0, XX = 1)
Running time 1500 m (s)	232.6 (10.7) 43.5%	234.2 (13.2) 39.1%	234.0 (15.1) 17.4%	*p* = 0.54	*p* = 0.81
Running time 3000 m (s)	509.6 (27.5) 37.2%	517.0 (28.0) 44.2%	518.1 (26.9) 18.6%	*p* = 0.08	*p* = 0.37
Running time 5000 m (s)	902.1 (61.7) 33.6%	912.2 (61.8) 46.2%	913.2 (59.5) 20.2%	*p* = 0.25	*p* = 0.58
Running time 10,000 m (s)	1860.9 (109.2) 34.8%	1885.7 (125.8) 42.9%	1889.3 (112.1) 22.4%	*p* = 0.22	*p* = 0.51
Running time marathon (s)	9148.6 (593.0) 30.4%	9220.7 (582.1) 47.6%	9129.1 (581.6) 22.0%	*p* = 0.94	*p* = 0.41
(b)					
*ACTN3*R577X females	RR *N* = 156 29.6%	RX *N* = 301 57.1%	XX *N* = 70 13.3%	Additive (RR = 0, RX = 1, XX = 2)	Recessive (RR = RX = 0, XX = 1)
Running time 1500 m (s)	269.4 (15.4) 34.7%	268.3 (14.0) 55.6%	262.6 (15.4) 9.7%	*p* = 0.35	*p* = 0.30
Running time 3000 m (s)	600.9 (44.7) 30.4%	602.8 (37.3) 61.6%	600.2 (48.5) 8.0%	*p* = 0.93	*p* = 0.89
Running time 5000 m (s)	1028.2 (79.1) 29.2%	1048.9 (97.1) 59.4%	1048.2 (88.9) 11.5%	*p* = 0.39	*p* = 0.83
Running time 10,000 m (s)	2067.6 (153.6) 27.7%	2101.0 (159.9) 57.4%	2067.6 (153.6) 14.9%	*p* = 0.61	*p* = 0.91
Running time marathon (s)	10,796.4 (818.2) 28%	10,667.3 (695.3) 54%	10,675.3 (552.8) 18%	*p* = 0.36	*p* = 0.78

All running times are expressed in seconds because statistical analyses were performed on times converted to seconds. The last two columns of the table correspond to the *p*-value of the linear regression, using an additive or a recessive genetic model. The percentage values represent the genotype proportions

We grouped the participants’ personal best times by event (1500, 3000, 5000, 10,000 m or marathon) as has been previously described [27]. First, we analysed the whole cohort of males and females separately. Then, we also performed a sub-analysis in males including only the endurance runners with times that were within 20% of the current world record of the examined events, following a similar approach to our recently published work [27]. We did not analyse the females in this sub-analysis because the sample size was too low (i.e. *n* < 5 for the XX genotype and *n* < 6 for the II genotype). We used the following world records as references:

- Male endurance runners. 3:26.00 in the 1500 m (Hicham El Guerrouj, Morocco), 12:37.35 in the 5000 m (Kenenisa Bekele, Ethiopa, 26:17.53 in the 10,000 m (Kenenisa Bekele, Ethiopia), 2:02:57 in the Marathon Dennis Kipruto Kimetto, Kenya);
- Female endurance runners. 3:50.07 in the 1500 m (Genzebe Dibaba, Ethiopia), 14:11.15 in the 5000 m (Tirunesh Dibaba, Ethiopia), 29:17.45 in the 10,000 m (Almaz Ayana, Ethiopia), 2:17:42 in the Marathon (Paula Radcliffe, UK).

### Genotyping

In the UK ~70% of the samples were collected as whole blood, ~20% as buccal swabs, ~10% as saliva. As has been previously described [17] blood was drawn from a superficial forearm vein into an EDTA tube and stored in sterile tubes at −20 °C until processing. Saliva samples were collected into Oragene DNA OG-500 collection tubes (DNA Genotek, Ottawa, Ontario, Canada) according to the manufacturer’s protocol and stored at room temperature until processing. Sterile buccal swabs (Omni swab; Whatman, Springfield Mill, UK) were rubbed against the buccal mucosa of the cheek for 30 s. Tips were ejected into sterile tubes and stored at −20 °C until processing. Genomic DNA was isolated from buccal epithelium, or white blood cells. In the UK DNA isolation was performed with the QIAamp DNA Blood Mini kit and standard spin column protocol, following the manufacturer’s instructions (Qiagen, West Sussex, UK). In brief, 200 μL of whole blood/saliva, or one buccal swab, was lysed and incubated, the DNA washed, and the eluate containing isolated DNA stored at 4 °C.

The Australian, Greek and Italian endurance runners’ DNA samples were genotyped using the polymerase chain reaction (PCR)-restriction fragment length polymorphism (RFLP) method as previously described [5]. The DNA samples of the UK, Polish and Russian endurance runners were genotyped in duplicates using an allelic discrimination assay on a Step One Real-Time PCR instrument (Applied Biosystems, Carlsbad, California, USA) with TaqMan® probes. To discriminate *ACTN3* R577X (rs1815739) and the *ACE* I/D alleles, a TaqMan® Pre-Designed SNP Genotyping Assay was used (assay ID: C_590093_1_ for rs1815739 (*ACTN3* R577X) and C__29403047_10 for rs4341 (a tag SNP in perfect linkage disequilibrium with the 287-bp *ACE* I/D in Caucasians [28])), including appropriate primers and fluorescently labelled (FAM and VIC) MGB™ probes to detect the alleles. For the genotyping of the UK samples the StepOnePlus and Chromo4 (Bio-Rad, Hertfordshire, UK) were used.

### Statistical analysis

To compare the endurance athletes’ running times between *ACTN3* R577X or *ACE* I/D genotypes, we converted the running times to seconds and initially used the one-way analysis of variance (ANOVA). Then a simple linear regression with running time as the dependent variable and genotypes as the independent variable was also applied. We used two genetic models: the additive model where RR = 0, RX = 1 and XX = 2, or DD = 0, ID = 1 and II = 2, and the recessive genetic model where RR = RX = 0 and XX = 1 or DD = ID = 0 and II = 1 as has been previously described [27]. Males and females were analysed separately. The level of significance was set at 0.05. All data analyses were conducted with the R statistical software with the lme4 and lrtest packages.

## Results

The mean (SD) personal best 1500 m, 3000 m, 5000 m, 10,000 m and marathon running times, according to the *ACTN3* and *ACE* genotype and distribution, are presented in Tables 1 and 2, respectively.

**Table 2:** Mean (SD) 1500 m, 3000 m, 5000 m, 10,000 m and marathon best running times in (a) males and (b) females in the three *ACE* I/D genotypes

(a)					
*ACE* I/D males	DD *N* = 314 32.4%	ID *N* = 452 46.6%	II *N* = 204 21.0%	Additive (DD = 0, ID = 1, II = 2)	Recessive (DD = ID = 0, II = 1)
Running time 1500 m (s)	233.3 (16.4) 29.9%	235.1 (11.6) 45.8%	235.7 (13.1) 24.3%	*p* = 0.50	*p* = 0.67
Running time 3000 m (s)	519.5 (28.0) 34.7%	517.7 (29.4) 43.7%	518.1 (24.7) 21.6%	*p* = 0.77	*p* = 0.93
Running time 5000 m (s)	914.1 (62.9) 31.7%	918.9 (60.8) 48.0%	916.1 (53.4) 20.3%	*p* = 0.80	*p* = 0.93
Running time 10,000 m (s)	1882.6 (101.3) 32.4%	1894.1 (116.6) 47.5%	1908.0 (132.1) 20.1%	*p* = 0.36	*p* = 0.45
Running time marathon (s)	9181.8 (665.1) 32.4%	9213.7 (549.0) 47.0%	9155.3 (491.5) 20.6%	*p* = 0.85	*p* = 0.57
(b)					
*ACE* I/D females	DD *N* = 127 28.4%	ID *N* = 229 51.2%	II *N* = 91 20.4%	Additive (DD = 0, ID = 1, II = 2)	Recessive (DD = ID = 0, II = 1)
Running time 1500 m (s)	268.7 (11.8) 37.5%	271.1 (18.8) 45.8%	263.9 (14.5) 16.7%	*p* = 0.65	*p* = 0.32
Running time 3000 m (s)	595.1 (31.2) 27.0%	612.9 (41.2) 53.9%	617.9 (40.2) 19.1%	*p* = 0.05	*p* = 0.30
Running time 5000 m (s)	1062.1 (65.0) 26.0%	1057.2 (103.0) 50.6%	1052.9 (95.9) 23.4%	*p* = 0.76	*p* = 0.81
Running time 10,000 m (s)	2140.8 (112.6) 30.6%	2090.4 (170.3) 52.8%	2095.1 (184.6) 16.7%	*p* = 0.48	*p* = 0.85
Running time marathon (s)	10,604.3 (560.9) 27.4%	10,765.9 (740.0) 51.3%	10,771.1 (707.8) 21.3%	*p* = 0.21	*p* = 0.61

All running times are expressed in seconds because statistical analyses were performed on times converted to seconds. The last two columns of the table correspond to the *p*-value of the linear regression, using an additive or a recessive genetic model. The percentage values represent the genotype proportions

ANOVA revealed no differences among the three genotypes (*p* > 0.05) of either *ACTN3* or *ACE* and running performance at any distance. Similarly, linear regression analysis using an additive or recessive genetic model also showed no association between running time and genotype of either genetic variant at any running distance (*p* > 0.05).

### No association between ACTN3 R577X or ACE I/D genotypes and personal best times in the whole cohort

**Fig. 1:**
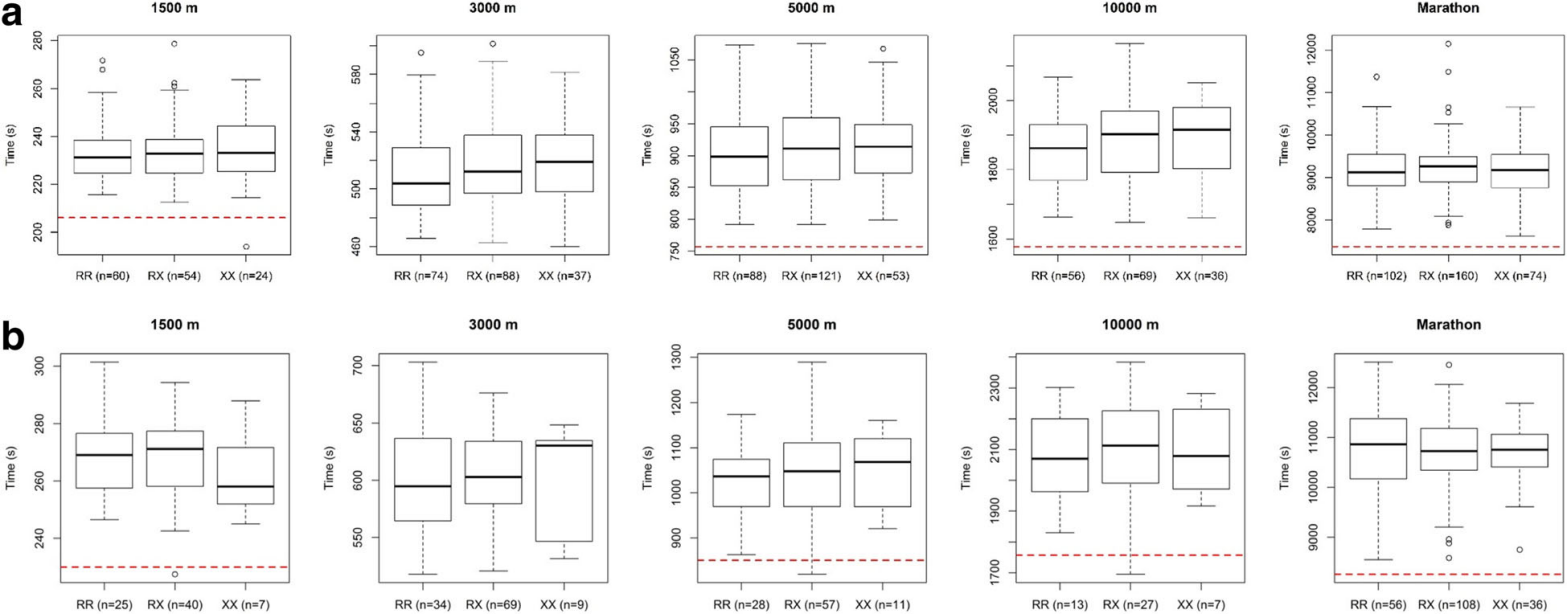
Individual 1500 m, 3000 m, 5000 m, 10,000 m personal best times in (**a**) male and (**b**) female endurance athletes according to their ACTN3 R577X genotype. Data are shown as boxplots and time is expressed in seconds. The red dashed line on each plot corresponds to the competition entry standard for the 2016 Olympic Games. 3000 m is not an Olympic event, so there is no red dashed line for this event

**Fig. 2:**
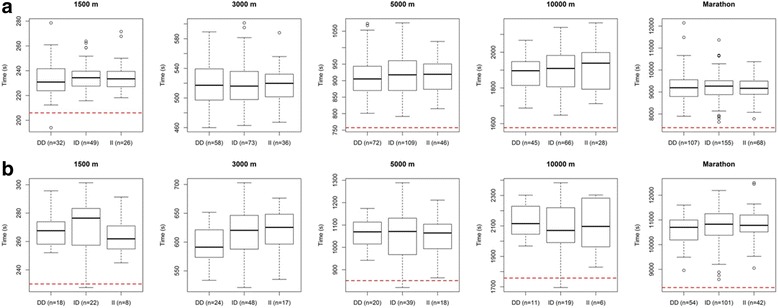
Individual 1500 m, 3000 m, 5000 m, 10,000 m personal best times in (**a**) male and (**b**) female endurance athletes according to their ACE I/D genotype. Data are shown as boxplots and time is expressed in seconds. The red dashed line on each plot corresponds to the competition entry standard for the 2016 Olympic Games. 3000 m is not an Olympic event, so there is no red dashed line for this event

In males and females alike and regardless of the chosen statistical analysis or genetic model, we found no association between either *ACTN3* R577X or *ACE* I/D genotype and 1500 m, 3000 m, 5000 m, 10,000 m or marathon personal best times (Figs. 1 and 2, Tables 1 and 2).

### No association between ACTN3 R577X or ACE I/D genotypes and personal best time in males within 20% of the world record

In males only, we conducted a sub-analysis of the athletes displaying times within 20% of the World record for the corresponding event, to see whether an association with the *ACTN3* R577X or *ACE* I/D variants could be detected at the high end of the performance spectrum. Regardless of the chosen statistical analysis or genetic model, we found no association between *ACTN3* R577X or *ACE* I/D genotypes and 1500 m, 3000 m, 5000 m, 10,000 m or marathon personal best time for those athletes within 20% of the world record (Table 3).

**Table 3:** Mean (SD) 1500 m, 5000 m, 10,000 m and marathon best running times in males within 20% of the world record in (a) the three *ACTN3* R577X genotypes and (b) the three *ACE* I/D genotypes

(a)					
*ACTN3*R577X males	RR *N* = 296 37.0%	RX *N* = 340 42.6%	XX *N* = 163 20.4%	Additive (RR = 0, RX = 1, XX = 2)	Recessive (RR = RX = 0, XX = 1)
Running time 1500 m (s)	230.8 (7.5) 44.2%	230.0 (8.1) 38.0%	228.7 (11.8) 17.8%	*p* = 0.34	*p* = 0.42
Running time 3000 m (s)	497.0 (16.2) 38.3%	502.2 (16.6) 42.8%	503.1 (18.8) 18.9%	*p* = 0.08	*p* = 0.37
Running time 5000 m (s)	857.5 (29.9) 34.6%	861.2 (30.2) 45.0%	861.7 (31.2) 20.4%	*p* = 0.52	*p* = 0.76
Running time 10,000 m (s)	1789.4 (66.0) 36.8%	1776.8 (66.6) 42.4%	1788.7 (70.7) 20.8%	*p* = 0.83	*p* = 0.76
Running time marathon (s)	8502.7 (330.0) 33.3%	8471.5 (302.1) 43.1%	8462.0 (337.8) 23.6%	*p* = 0.64	*p* = 0.76
(b)					
*ACE* I/Dmales	DD N = 229 33.7%	ID *N* = 306 45.0%	II *N* = 145 21.3%	Additive (DD = 0, ID = 1, II = 2)	Recessive (DD = ID = 0, II = 1)
Running time 1500 m (s)	228.1 (11.0) 29.2%	231.6 (8.0) 46.9%	232.1 (8.2) 24.0%	*p* = 0.11	*p* = 0.39
Running time 3000 m (s)	501.8 (16.9) 34.0%	502.1 (18.3) 43.3%	505.3 (17.0) 22.7%	*p* = 0.49	*p* = 0.42
Running time 5000 m (s)	866.9 (29.2) 34.4%	861.5 (33.7) 45.9%	858.6 (29.2) 19.7%	*p* = 0.32	*p* = 0.52
Running time 10,000 m (s)	1795.5 (66.8) 35.5%	1789.8 (73.2) 47.7%	1786.4 (64.8) 16.8%	*p* = 0.70	*p* = 0.79
Running time marathon (s)	8495.1 (287.9) 34.1%	8507.6 (343.5) 42.9%	8457.5 (327.6) 22.9%	*p* = 0.79	*p* = 0.64

All running times are expressed in seconds because statistics were performed on times converted to seconds. The last two columns of the table correspond to the *p*-value of the linear regression, using an additive or a recessive genetic model. The percentage values represent the genotype proportions

## Discussion

Here, we have utilised similar approach as we previously did in elite sprinters [27] in a large cohort of elite Caucasian endurance runners. This quantitative assessment of genotype with qualifying time in 1064 personal best times of 698 elite endurance runners suggests that the potential association between *ACTN3* R577X and *ACE* I/D genotypes and elite endurance running time is unproven.

In the present study, we examined whether a genotype association existed within athletes competing in particular endurance-running events (1500 m, 3000 m, 5000 m, 10,000 m and marathon) and in a subset of high-level athletes with personal-best times within 20% of the World record. Previous reports have grouped together endurance athletes from mixed endurance sports disciplines and events without quantifying measures of their actual endurance performance [6, 8–12]. Here, we have embraced a more stringent approach and included only endurance runners whose main sporting discipline was the 1500, 3000, 5000, 10,000 m or marathon, including their personal-best running performance. In this manner, we were able to differentiate between events that are estimated to have a different energy reliance on the aerobic system ranging from 77 to 86% (1500 m), 86-94% (3000 m) whereby it becomes increasingly dependent on aerobic metabolism up to the Marathon [29]. Despite addressing these subtle performance requirement differences, our results suggest that neither the *ACTN3* R577X nor *ACE* I/D polymorphisms are likely to influence Caucasian endurance runners’ personal best times in 1500, 3000, 5000, 10,000 m and marathon, even at the high end of the performance spectrum.

The *Actn3* KO mouse model attempted to mimic the *ACTN3* R577X polymorphism in humans. Metabolically, the KO mice show a higher activity of oxidative enzymes and a lower activity of enzymes involved in the anaerobic pathway [30]. In addition, KO mice show enhanced glycogen accumulation due to lower glycogen phosphorylase activity [30, 31]. Their fast skeletal muscle fibre properties shift towards a slower metabolic profile, which has been linked to an increase in calcineurin signalling activity [32] and theoretically can favour endurance performance. Top-level endurance running performance is considered to be predominately based on the metabolic profile of slow-twitch (type I) and some recruitment of intermediate (type IIa) fibres due to the high reliance on the aerobic energy system, which has been hypothesized to favour the 577XX genotype. However, it may also depend on the endurance runner’s ability to recruit a greater number of type IIa myofibres during tactical surges (competitively critical phases requiring increase in pace) or finishing stages of a race (a sprint over a short distance), both of which require an increase in anaerobic energy/muscle recruitment (and may favour the 577RR genotype). While it is difficult to determine the relative contribution of muscle fibres in human competitive performance, it is well understood that murine muscle contains a significantly higher percentage of myofibres with faster twitch properties than human muscle and any association with the presence/absence of α-actinin-3 protein would probably be enhanced in this model. Therefore, any such association in humans might be extremely limited, thus offering no tangible or detectable advantage to a competitive α-actinin-3 deficient (*ACTN3* 577XX) endurance runner. In our study, we included only Caucasian endurance athletes because thus far we have recruited insufficient individuals of other ethnicities for effective analysis, so we cannot rule out the possibility that an association exists between *ACTN3* R577X or *ACE* I/D and endurance running performance in elite runners with different geographic ancestry. However, most published associations on which our original hypothesis was based involved athletes or other individuals who were Caucasians.

Endurance performance is considered to be a complex trait effected by both genetic and environment (training) [33]. As recently shown, not only metabolism but also anthropometric and biomechanical factors are important in determining elite performance success [34]. Here we have not found evidence that supports the involvement of the two gene variants we studied in endurance running performance. Endurance running performance is dependent on extensive training and there is little evidence of either *ACTN3* R577X or *ACE* I/D being associated with training responses of aerobic parameters [35]. Interestingly, in addition to this, it has been suggested that epigenetic modifications related to the *ACE* gene may also have a part to play in these discrepant findings [36]. Indeed, epigenetic factors such as CpG islands that modify the expression of genes without alteration to the DNA coding sequence have been identified in the *ACE* gene promoter [37]. Therefore, future studies on the *ACE* gene, in relation to human endurance, could benefit if these epigenetic factors that regulate ACE expression [37] were considered in addition to the I/D genotype [36]. These epigenetic factors reported to influence *ACE* activity cannot be controlled by our quantitative approach and sophisticated experimental designs are required to control more external factors and possibly explain some of the discrepant findings between *ACTN3* R577X and *ACE* I/D genotype and endurance phenotypes.

The multi-centre cohort we used is larger than any previous such study regarding endurance performance, but a very small effect size could still go undetected using our sample size. It is increasingly recognised that the reality of complex human biology is that inter-individual differences in endurance performance are expected to be influenced by many common and perhaps rare genetic variations; none of which have been discovered at a genome-wide significance level or consistently replicated [3].

Our study of quantitative measures of endurance performance in a large homogeneous group of elite endurance runners suggests that the potential association between *ACTN3 R577X* and *ACE* I/D genotypes and elite endurance running performance should be regarded as unproven. Large population studies are needed to detect significant proportions of the underlying genetic profile and biology contributing to endurance performance.

## Conclusions

In conclusion, this study has presented evidence that the *ACTN3* and *ACE* polymorphisms are not associated with running performance in elite athletes, contrary to our hypotheses and again exposing the fallacy of products offered by numerous direct-to-consumer (DTC) genetic testing companies [38]. Our understanding of the genetic influences on human physical performance is evolving rapidly in the postgenomic era. Much more work remains to be done to answer a number of major questions in the field. One study utilising a genome-wide approach has been published recently [3] and future studies investigating the genomic contribution to elite endurance performance using genome-wide and targeted sequencing approaches are still needed to discover more genetic variants contributing to human physical performance capability. Understanding both genetic and environmental contributions, and how they interact, will be beneficial in understanding elite performance and muscle function in sport, health and disease.

## Abbreviations

DTC: Genetic testing companies: Direct-to-Consumer genetic testing companies
GWAS: Genome-Wide Association Study
